# What Is a Good Death in South Asia? A Systematic Review and Narrative Synthesis

**DOI:** 10.1111/jnu.70002

**Published:** 2025-03-18

**Authors:** Lihini Wijeyaratne, Odette Spruijt, Saroj Jayasinghe, Sumit Kane, Udayangani Ramadasa, Jennifer Philip

**Affiliations:** ^1^ Faculty of Medicine, Dentistry and Health Sciences University of Melbourne Carlton Victoria Australia; ^2^ Tasmania North Specialist Palliative Care Service Hobart Tasmania Australia; ^3^ Faculty of Medicine University of Colombo Colombo 8 Sri Lanka; ^4^ Department of Medicine, Faculty of Medicine Sabaragamuwa University of Sri Lanka Ratnapura Sri Lanka; ^5^ Melbourne School of Population and Global Health Nossal Institute for Global Health, The University of Melbourne Victoria Australia; ^6^ St Vincent's Hospital Fitzroy Victoria Australia

**Keywords:** caregivers, good death, healthcare workers, palliative care, patients, review, South Asia

## Abstract

**Introduction:**

To deliver palliative care, it is important to understand what a “good death” means to the relevant people. Such studies have mostly occurred in high‐income settings that usually live by Western ideals. What matters to people is likely to vary across different regions of the world, influenced by multiple factors. Although there is a great need for palliative care in South Asia, there is a lack of comprehensive understanding of what a good death means in this setting. This study aimed to increase understanding of what is considered a good death in South Asia.

**Design:**

Systematic review and narrative synthesis.

**Method:**

A systematic search was conducted across eight databases, an Advanced Google search, and a bibliography search of selected articles. A data‐based convergent synthesis was performed, along with quality appraisal.

**Results:**

Twenty‐five empirical studies were selected for analysis from India, Pakistan, Bangladesh, Sri Lanka, and Bhutan. Four themes emerged. *Mutual care and connection support a continued sense of self*: contributing to others, while receiving connection through relationships and spiritual practices, was important for patients and supported by families and healthcare workers. *Freedom to choose—privilege or burden*?: the choice to participate in care was necessary for some patients but a burden for others, who preferred the family to lead their care. Severe uncontrolled pain and financial distress precluded choice for some patients, who felt death was the only option. Decisions regarding artificial prolongation of life were complex for patients and healthcare workers. *Opportunities in the last days*: when actively dying, there was general agreement on the importance of being pain‐free, feeling safe, and having family present. Home was not always the preferred place of death. For family, it was critical to perform last rites. *After death matters*: What happens after death—influenced by leaving a legacy and religious beliefs—affected all parties before, during, and post‐death.

**Conclusions:**

To our knowledge, this is the first review of what a good death means in South Asia. There is a dearth of research from most South Asian countries. Although the South Asian perspective has similarities with the Western perspective, we note important nuances around decision‐making, prolongation of life, prognostic awareness, and wanting to end one's life, moderated by culture, religion, and poverty. We support policies that account for these variations. Ongoing work is required to provide good symptom management, thus increasing opportunities for patient participation in care. Further research is needed in areas of ethics and religion at the end of life in South Asia.

## 1 Introduction

The majority of deaths globally occur in low‐income and middle‐income countries (LMICs). It is estimated that, in 2015, 81% of deaths associated with serious health‐related suffering (SHS) occurred in these countries, the commonest causative disease being cancer. Physical symptoms, mainly pain, accounted for the majority of this suffering (Knaul et al. 2018).

In South Asia (India, Sri Lanka, Bangladesh, Nepal, Bhutan, Maldives, Pakistan and Afghanistan), approximately 9.9 million people are estimated to be experiencing SHS (Knaul et al. 2018) Of the estimated 1.92 billion people living in South Asia, 36% are at risk of catastrophic healthcare expenditure for surgical care; that is, when out‐of‐pocket spending for healthcare by a household exceeds a predefined proportion of a household's capacity to do so, resulting in an inability to obtain basic needs without using savings, borrowing money, or selling assets (Nambiar et al. 2021).

Palliative care aims to improve quality of life for patients experiencing SHS due to serious illness and their families (Radbruch et al. 2020). However, despite 80% of palliative care needs existing in low–middle‐income countries, it is estimated that only 30% of palliative care services operate in these regions (Connor et al. 2021). South Asia faces multiple challenges in terms of palliative care delivery, such as limited access to opioids and a lack of policies and legislation to support the development of palliative care services in the region. The latter contributes to potentially inappropriate healthcare practices at the end of life (Nambiar et al. 2021). Despite the substantial estimated need for palliative care in the region (Connor 2020; Singh and Harding 2015), palliative care provision is isolated at best (Connor 2020).

The holistic approach of palliative care involves the identification and management of distress in its various forms, while aligning with the goals, values, and beliefs of patients and their families (Radbruch et al. 2020). Values of patients, family caregivers, and healthcare professionals can differ and influence the nature of palliative care that is delivered (Steinhauser et al. 2001). Given the integral nature of identifying values and preferences in the setting of serious illness, significant attention has been devoted to attempt to define what a “good death” means to patients, family caregivers, and healthcare professionals. (Kastbom et al. 2017; Krikorian et al. 2020; Sepulveda et al. 2022; Zaman et al. 2021).

Many of these studies have focused on people living in Western settings (Zaman et al. 2021). Despite the commonalities that exist in what people may consider important at the end of life, such as symptom control and involvement of family, (Hirai et al. 2006; Ibrahim and Harhara 2022; Soto‐Perez‐de‐Celis et al. 2016), it is likely that there is also variation across countries and cultures. The importance of spirituality and religion is one such difference (Gafaar et al. 2020). The conception of autonomy has also been noted to differ across various contexts (Dutta et al. 2020). Cultural differences have also been found to affect communication preferences, care preferences, decision‐making, and the perception of suffering at the end of life (Cain et al. 2018).

There are no reviews of what is considered a good death in South Asia. To create palliative care services that are feasible and relevant to South Asia, it is necessary to have an understanding of what matters to people living in these regions of the world. This may provide greater clarity on how and where to direct valuable limited resources within palliative care.

This study aimed to increase understanding of what is considered a good death in South Asia. By doing so, this study will provide an understanding of what is valued at the end of life for people living in South Asia and the commonalities and differences that may exist compared to those living in Western countries. This understanding may form the basis for establishing palliative care services that best meet the needs of South Asian populations.

## 2 Design

A systematic review with narrative synthesis was conducted, informed by the Guidance on the Conduct of Narrative Synthesis in Systematic Reviews (Popay et al. 2006). The review is reported as per the Preferred Reporting Items for Systematic Reviews and Meta‐Analyses (PRISMA) guideline (Page et al. 2021).

This review aimed to explore the values and preferences regarding end‐of‐life from the perspectives of patients, families, healthcare workers, and any other community members in South Asia through the lens of a “good death.”

## 3 Materials and Methods

Inclusion and exclusion criteria are detailed in Table 1.

**TABLE 1:** Inclusion and exclusion criteria.

Inclusion criteria	Exclusion criteria
Empirical studies, i.e., qualitative, quantitative, or mixed‐method studies	Non‐empirical studies—conference abstracts, conference reports, reviews, books, book chapters, book reviews, letters, editorials, case reports, case series, dissertations
From South Asian region (Sri Lanka, India, Nepal, Bangladesh, Bhutan, Pakistan, Afghanistan, Maldives) as per World Bank (World Bank 2023)	Focus on attitudes to death, coping with death, knowledge of palliative care, euthanasia/assisted dying, evaluation of assessment tools for quality of death and dying, studies on South Asians not living in country of origin
Focused on or described a “good death,” preferences, experiences of any adult stakeholder, e.g., patient, healthcare worker, caregivers, public	Pediatric palliative care

### 3.1 Data Sources and Search Strategy

Databases searched were Ovid MEDLINE(R) and Epub Ahead of Print, In‐Process, In‐Data‐Review & Other Non‐Indexed Citations and Daily, APA PsychInfo (Ovid), Embase Classic + Embase (Ovid), Global Health (Ovid), CINAHL Complete (EBSCOhost), Scopus (Elsevier), Web of Science (Clarivate), and Google Scholar.

The search was run on April 26, 2023 and combined both text terms and subject headings for “good death” and “South Asia.” No date or language limits were applied.

The search strategy for Ovid Medline and other databases is provided in Data S1.

A search strategy was developed in conjunction with a medical librarian. Key phrases for “good death,” “South Asia,” “palliative care,” “cancer,” “attitude to death,” “communication,” and “preferences” were brainstormed and trialed on Ovid Medline to refine the relevant search terms and strategies. Key review and research articles were also used to delineate the search strategy. The final search strategy was developed in Ovid Medline without date or language limits. MeSH terms or the equivalent, if available, were included in all database searches. The EPOC filter was adapted to create the search strategy for “South Asia” (Cochrane Effective Practice and Organisation of Care (EPOC) 2022), LMIC filters. The overall search strategy was adapted for the other databases (for subject headings and search syntax).

An advanced Google search was also conducted, as a means of including any further empirical research papers that were not included in the database searches. This was run using the terms “good death” for each country, for example, “good death Bhutan,” on June 9, 2023. All results from all pages resulting from the searches were screened based on their titles. Titles that seemed relevant were followed up by visiting the webpage. The same inclusion and exclusion were applied as for the database searches.

Bibliographies of the final selected studies from the database and advanced Google searches were also hand‐searched.

### 3.2 Study Selection

Results from the searches were downloaded to EndNote bibliographic management software (Version 20) (The Endnote Team 2013) and subsequently uploaded to Covidence for deduplication and screening (Covidence 2023). Screening was done as per the inclusion and exclusion criteria by the principal investigator LW. Following the completion of title and abstract screening stages, studies eligible for full‐text review were separately evaluated by two investigators LW and JP. Any disagreements were resolved through adjudication by a third investigator OS. The same process was used once relevant bibliographies of selected studies were screened.

### 3.3 Data Collection and Analysis

The author LW tabulated the following information from selected articles: authors, journal, year of publication, aims, country/setting, participants, study design, and main results.

A narrative synthesis was conducted using a data‐based convergent design to enable the synthesis of the varied study types (Hong et al. 2017). Data were extracted and analyzed from the perspectives of patients, healthcare workers, family caregivers, and the public. Qualitative studies were analyzed using thematic analysis. One investigator LW repeatedly read the studies and extracted initial codes based on the findings and data from the studies, which were then grouped into themes. Data from quantitative studies were tabulated and grouped according to common topics across these studies (Popay et al. 2006). The main points were then integrated into the existing framework created by thematic analysis of qualitative data. Idea webbing and concept mapping were undertaken. The overall themes were discussed with all investigators from multiple perspectives to refine ideas until the final narrative synthesis resulted. Any disagreements were resolved through discussion between all the authors. The final common rubric is presented below.

### 3.4 Quality Appraisal

Quality appraisal was conducted by two investigators LW and JP using validated tools for each study type. In line with Cochrane guidance (Noyes et al. 2018), tools were used to assist with understanding the strengths and limitations of studies and how these may affect data synthesis. The Critical Appraisal Skills Program (CASP) tool was used to appraise qualitative studies (Critical Appraisal Skills Programme (2022), CASP Qualitative Studies Checklist [online]) and the Mixed Method Assessment Tool (MMAT) (Quan Nha Hong et al. 2018) for mixed‐method studies. For quantitative studies, we modified the assessment tool (Hoy et al. 2012) by excluding questions 6 (acceptable case definition) and 9 (appropriate period prevalence) as these could not be applied in the absence of a clear definition of a “good death” (Zaman et al. 2021) and they were felt to not be necessary to satisfactorily answer the research question. A discrete choice experiment study was evaluated as per the International Society for Pharmacoeconomics and Outcomes Research (ISPOR) checklist for conjoint analysis (Bridges et al. 2011).

Given the limitations of the quality appraisal tools used (Long et al. 2020), the subjectivity involved when appraising quality (Sandelowski 2015) and the recommendations against using scoring systems to grade the quality of studies (Hoy et al. 2012; Noyes et al. 2018), it was determined that an appreciation of the common methodological issues encountered during the quality appraisal process would be of most relevance. No studies were excluded based on quality appraisal.

## 4 Results

**FIGURE 1:**
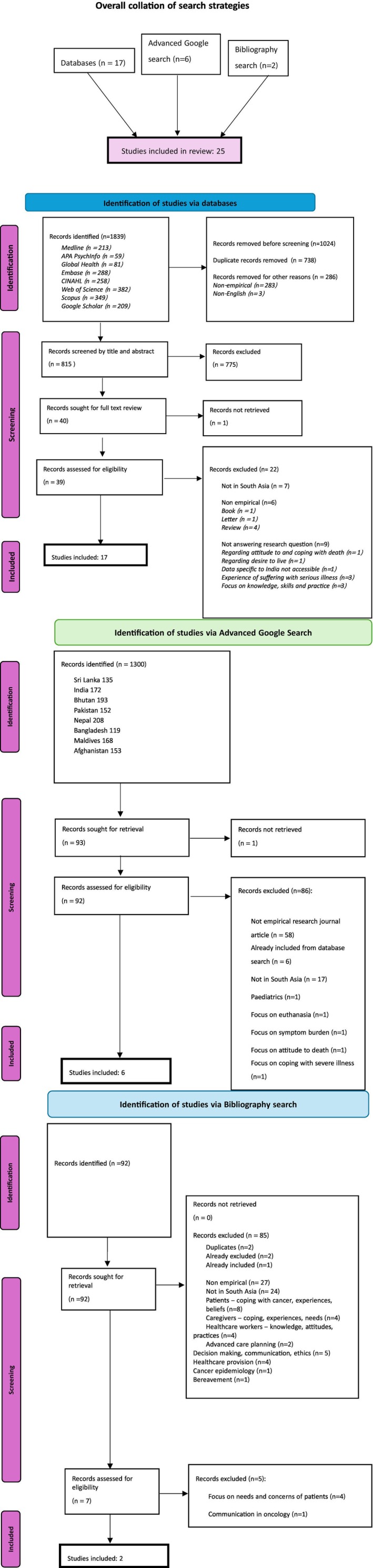
Search strategies and PRISMA flowcharts.

In total, 25 studies were selected for analysis. Figure 1 shows the search strategies and PRISMA flowcharts for the database search, advanced Google search, and bibliography search. The database search identified 1839 articles. Three articles were non‐English (French, German, and Spanish) and were therefore excluded at this stage. Of 815 articles that were screened, 775 were excluded and 39 were assessed for eligibility. Seventeen studies were included. Advanced Google searching yielded 92 records suitable for assessing eligibility, of which six were included. Searching bibliographies of selected articles resulted in the identification of two further articles. Reasons for the exclusion of studies are detailed within the flowcharts.

Study descriptors and key findings are presented according to the country of origin and the included participants (Table 2). The majority of studies were conducted in India (*n* = 17), with smaller numbers from Pakistan (*n* = 4), Sri Lanka (*n* = 2), Bangladesh, and Bhutan (both *n* = 1). Fourteen studies were qualitative, 10 were quantitative (9 surveys, 1 discrete choice experiment), and one was a mixed‐method study. Patients with advanced cancer were the most studied group, with other participants being caregivers, healthcare staff (mostly doctors and nurses), and members of the public (community members, spiritual leaders, crematorium staff). Religions of participants included Hinduism, Islam, Christianity, Tibetan Buddhism, and Theravāda Buddhism.

**TABLE 2:** Study descriptors and key findings.

Author, journal, year	Research aims	Setting/country	Participants	Study design, methodology	Key findings
India					
India‐Patients					
(Raju and Krishna Reddy 2018) Indian Journal of Palliative Care 2018	To explore concerns regarding death among inpatients with glioblastoma	Department of Neurosurgery, National Institute of Mental Health and Neurosciences, Bengaluru, India	31 inpatients with glioblastoma awaiting neurosurgery	Qualitative Interviews	Poor understanding of illness, desire for more information Fear, sadness, not wanting to be a burden, wanting to die at home Drew upon family relationships and spirituality to manage anxiety related to death Preparing for death was important
(Sepulveda et al. 2022) Journal of Pain and Symptom Management 2022	To quantify preference weights for key indicators relevant to end of life care from patient perspective	India, Kenya, Singapore, UK, USA	Proxy bereaved caregiver for close friend or family member in last 2 years (*n* = 250)	Quantitative, discrete choice experiment 13 indicators based on scoping review and expert opinion Online survey of bereaved caregivers' perceptions of patient priorities using vignettes regarding last 6 weeks of life	Nearly half the respondents from India and the US had a bachelor's degree or higher education degree, whereas less than 20% of participants had this level of education in Singapore, Kenya, and the UK. Compared to US, caregivers from India were more likely to leave decisions to doctor, believe health depends mostly on luck, be younger, with poorer end of life experience or be recently bereaved. Prioritization of top 5 indicators in Indian cohort: Manage pain and discomfort Quality of life extending treatments Clean and safe space Asked enough questions Clear and timely information The relative importance of the patient dying in the preferred setting was nearly twice as important in the US as in India (7.7% vs. 4%). UK participants ranked spiritual needs with almost half the importance of those from India, the US, and Kenya (3.1% vs. 5.7%–5.9%).
(Wajid et al. 2021) BMC palliative care 2021	To explore the benefits of hospice care for terminally ill patients with cancer	Karunashraya Hospice, Bengaluru, India	Hospice patients with terminal cancer (*n* = 8)	Qualitative interviews	Importance of pain management, a lack of which would induce suicidality; importance of contributing to others; to die without pain in a peaceful place without burdening others; to be treated with compassion and not burden family
(Pany et al. 2018) Indian Journal of Palliative Care 2018	To explore preferences of elderly people regarding end‐of‐life decision‐making and social welfare measures	Odisha, India	Patients attending geriatric clinic—8.7% had a terminal illness (generally advanced cancer) (*n* = 138)	Quantitative Survey	64% wanted to die at home with family present (usually spouse and children) 32% wanted to die in hospital with all medical care provided 64% wanted a senile death instead of a hasty death
(Chittem et al. 2022) Supportive Care in Cancer 2022	To understand perspectives of Indian cancer patients regarding end of life and how these influence end‐of‐life treatment preferences	Cancer Hospital, Hyderabad, India	Patients with advanced cancer (*n* = 25)	Qualitative Interviews	Importance of: – completing family responsibilities, – duty to family (to die as a *sumangali*), – to maintain hope and find a cure, – to have symptoms controlled, – to have access to euthanasia due to pain causing loss of function and distress. – Faith in God was a source of strength, and control was given to God regarding the future
(Earnest and Gnanadurai 2022) Indian Journal of Continuing Nursing Education 2022	To understand what a good death means to terminally ill patients	Hospice, Chennai, Tamil Nadu, India	Terminally ill patients with cancer (*n* = 120)	Quantitative Survey	Most important for a good death were pain control, dying during sleep, dying when loved ones were present and after completing responsibilities
(Simha et al. 2013) Indian Journal of Palliative Care 2013	To explore the spiritual concerns of patients with cancer receiving palliative care in a hospice setting in India	Karunashraya Cancer hospice, Bangalore, India	Cancer patients (*n* = 10)	Qualitative Interviews	Acts of completion, participating in spiritual practices, maintaining faith in God and addressing karma were important to patients.
(Devakirubai and Gnanadurai 2013) Asian Journal of Nursing Education and Research 2013	To explore end‐of‐life care preferences among terminally ill patients	Jeevodhaya Hospice, Chennai, Tamil Nadu, India	Patients with advanced cancer (*n* = 10)	Quantitative Modified pilot survey	7/10 wanted spouse to initiate conversation regarding end‐of‐life wishes All patients preferred to die earlier than continue living and suffer. 6/10 did not want artificial hydration, nutrition, or cardiopulmonary resuscitation 7/10 did not want new treatments If having capacity, 7/10 patients wanted to make their own decisions, and 6/10 wanted healthcare workers to make decisions if they lost capacity 8/10 wanted to talk about spiritual needs and advanced care wishes 9/10 wanted to die at home, half wanted to die in the presence of family
India‐Caregivers					
(Sudhakar et al. 2021) Psycho‐Oncology 2020	To explore experiences of bereaved caregivers of patients with advanced cancer, regarding quality of death	Cancer Institute, Chennai, Tamil Nadu, India	Recently bereaved caregivers (*n* = 108) Bereaved for 1 week	Qualitative Telephone interviews regarding the deceased relative's experience in their last week of life	Poor symptom control caused distress Patients reacted differently to prognostic awareness (preparation vs. distress) and unawareness (hope vs. uncertainty). Most patients did not know prognosis but likely suspected the truth. Family tried to protect patients from the truth and reduce distress.
(Ramalingam and Ganesan 2019) Indian Journal of Palliative Care 2019	To explore end‐of‐life care practices in Tamil Nadu	Rural Coimbatore, Tamil Nadu, India	Person with experience of caregiving for elderly or terminally ill person in household (*n* = not available)	Qualitative 2 focus group discussions 4 interviews	Rituals in the community for a peaceful death—oil bathing, feeding with holy water or milk (including with reduced consciousness) to end suffering, feeding water and soil or mud Reasons for rituals – Financial—cost of hospitalization, hospital cannot provide care – Cultural—will satisfy the deceased soul with a peaceful death – Moral duty of family – Spiritual—to reach God and deceased ancestors
India‐Healthcare Professionals					
(Pinto et al. 2019) Indian Journal of Palliative Care 2019	To increase understanding of psychological and interpersonal needs of people at the end of life, through the perspective of healthcare providers	Karunashraya Palliative Care, Bengaluru and Ave Maria Palliative Care, Mangalore, India	11 healthcare workers in palliative care centers (6 counselors, 2 physicians, 2 social workers, 1 nurse)	Qualitative 1 focus group discussion 5 interviews	Psychological needs: different reactions to mortality and spirituality, experience of guilt, anger, anxiety, depression Interpersonal needs: need for time with family and spouse, need for detachment, experience of rejection, connection with healthcare workers Need for completion and preparation—saying goodbye, addressing responsibilities, reconciling, reflecting on life
(Doorenbos et al. 2006) International Nursing Review 2006	To describe dignified dying and nursing actions used to promote this, and evaluate survey tool	India–Mumbai (government hospitals), North (private hospitals) and South (private Christian hospitals)	Nurses with experience of caring for dying inpatients (*n* = 229)	Quantitative Dignified dying survey tool	Illness‐related actions—symptom control, clear communication to enable preparation and maintain hope, encourage discussing feelings Measures to maintain dignity–spiritual support (North—Hindu rituals and prayers; South—praying, getting a pastor); maintain pride and self‐esteem by spending time, showing interest and providing physical touch Social dignity‐related actions—involving family in care, respecting the patient
(Coenen et al. 2007) Oncology Nursing Forum 2007	To investigate nursing interventions in Ethiopia, Kenya, India, and USA to promote dignified dying	India Kenya Ethiopia USA	Nurses who had experience carrying for dying patients (Indian nurses *n* = 229)	Qualitative Thematic analysis of written answer to question which was part of the dignified dying survey	Results for India: Illness‐related interventions: to reduce pain and promote comfort (analgesia, giving oxygen, yoga meditation, holistic care, bathing, positioning) Psychological and spiritual support: providing presence, maintain hope and faith, fulfill last wishes, praying, chanting, spiritual practices Social dignity: involving family, creating peaceful environment, singing songs, listening, tender loving care, provide touch
India‐Public					
(Kulkarni et al. 2014) Indian Journal of Palliative Care 2014	To investigate preferred place of death for people living in Pune	Pune, India	Public (*n* = 3440)	Quantitative Survey	83% preferred to die at home
India‐Mixed					
(Namdul 2021) Religions 2021	To explore meaning and perception of death through the lens of Tibetan Buddhist culture	Tibetan refugee communities in Southern India	Tibetan refugee communities, bereaved caregivers healthcare workers, spiritual leaders (*n* = not stated)	Qualitative 18‐month ethnographic study	To die peacefully without pain, fear, or regret To die with compassion, and good karma, with confidence for next life State of mind matters at time of death, an opportunity to transcend the self To be prepared for death regardless of time and location To have awareness, hear scripts from Tibetan Book of the Dead
(Chacko et al. 2014) International Journal of Palliative Nursing 2014	To explore perspectives of patients with advanced cancer and healthcare workers on end of life	Inpatient and outpatient radiation oncology department, Christian Medical College, Vellore, India	Patients with advanced cancer (*n* = 140) Healthcare workers—doctors and nurses (*n* = 40)	Quantitative Modified surveys	Patients: important to talk about quality of life with health team, discuss fear, receive emotional support, know prognosis, family to be involved in care, support for family, engage in spiritual acts, to die at home, be comfortable. Prolongation of life had mixed opinions. 90% felt finances were extremely important—a lack of money led to feeling burdensome, contemplating suicide and being unable to continue with treatments. A frequently encountered comment was that clinicians revealed the prognosis to the caregiver but not to the patient, as it may unfavorable affect the patient psychologically Healthcare workers: important to manage symptoms, provide information, provide emotional support, and support spiritual needs. Mixed opinions about artificial prolongation of life
(Ghosh and Bk 2024) OMEGA‐ Journal of Death and Dying 2022	To understand impact of COVID‐19 on mourning practices of Hindu communities in Kerala and West Bengal in context of COVID‐19 pandemic	Palakkad crematorium, Kolkata crematorium Palakkad and Kolkata hospital	Hospital staff, health officials, nursing staff, priests, bereaved relatives, crematorium staff (*n* = 15)	Qualitative Ethnographic‐documentary evidence, observation, unstructured narratives, interviews. Consolidated narrative data with newspaper articles and other documentary evidence	A good death involves completion of post‐death rituals; relatives being aware of patient's impending death and being able to honor wishes of the deceased; to hear *Bhagavad Gita* gives dignity in death
Pakistan					
Pakistan‐Patients					
(Zafar et al. 2016) Palliative medicine 2016	To evaluate preferences of cancer patients regarding disclosure of disease status, palliative care, and end‐of‐life care	Shaukat Khanum Memorial Cancer Hospital & Research Centre, Lahore, Pakistan	Patients with cancer of all stages (*n* = 520 for 1st survey +100 for re‐test = 620)	Quantitative Modified survey	60% of patients wanted detailed information about prognosis and most wanted family to know as well Over half did not want artificial prolongation of life, yet wanted all possible treatments until death 60% wanted psychology support for emotional problems 42% wanted to die at home (19% after 3 months) Most important: Spiritual and religious wellbeing Not being a burden Relationships with family and friends Pain and symptom management Sense of control and dignity Avoid inappropriate prolongation of dying
(Nadeem et al. 2017) Pakistan Armed Forces Medical Journal 2017	To understand what a good death is among Muslim patients with cancer and their healthcare providers in Pakistan compared to The Future of Health and Care of Older People report	Combined Military Hospital, Rawalpindi, Pakistan	Inpatients with cancer (*n* = 55) Healthcare workers caring for patients with cancer—doctors, nurses, paramedical staff (*n* = 55)	Sequential Mixed methods Modified questionnaire Interviews	Important to patients and healthcare workers were aspects regarding time of death; control over treatments; dignity and privacy; where death occurs and who is present; spiritual and emotional support; leave a will for the bereaved; complete unfinished business; and secure family financially and socially.
(Saeed et al. 2020) Journal of Pain and Symptom Management 2020	To explore decision‐making, attitudes to end of life and knowledge of palliative care among Pakistani patients on dialysis	Dialysis units in Punjab (Rawalpindi, Sialkot, and Lahore) and Sindh province (Hyderabad), Pakistan	Patients on dialysis (*n* = 522)	Quantitative Modified dialysis survey tool	68% wanted more information about illness, 54% wanted more information regarding prognosis, 61% wanted information on different management including symptom management and withdrawal of dialysis 63% of patients considered it important to prepare and plan for death, but only 18% were able to discuss these issues with their doctors Approximately two‐thirds wanted family to be involved in making decisions, half wanted nephrologist to discuss quality of life and address spiritual, psychological and social issues. Nearly two‐thirds of patients were comfortable discussing end‐of‐life issues with their physician and family 54% had started dialysis because of physician's wish, and 62% wished they never had. 47% wanted to focus on quality of life, 19% wanted to prolong life, 34% were unsure. 56% wanted full resuscitation. Nearly one‐third wanted to discuss end‐of‐life issues with specialist, but 56% wanted to do so only when need arose 68% preferred to die at home
Pakistan‐Mixed					
(Ali 2020) Frontiers in Sociology 2021	To compare death rituals between ordinary times and during the COVID‐19 pandemic	Pakistan	Members of public and bereaved family members of patients with COVID‐19	Qualitative Autoethnographic and ethnographic Content and document analyses	An ideal death would include: – dying a natural death in old age; – Dying at home; – if married, dying at husband's or son's home; – in the presence of family who would perform all rituals such as ablution of the body, dressing the body in a *kafan* and carrying it on a *charpoy*; and that – the family would perform the *Namaz‐e‐Janaza* (funeral prayer).
Bangladesh					
Bangladesh‐Public					
(Joarder et al. 2014) Journal of Cross Cultural Gerontology 2014	To explore meaning of death and the impact of this elderly people in Bangladesh village	Kakabo, Bangladesh	Community elders—5 males, 3 females	Qualitative 7 interviews, Informal discussions, observing daily routines, Body mapping	A good death is peaceful, without suffering, in the presence of family. A good death is given by God, and karma is a strong influence on whether one has a good or bad death Important to be physically well and independent—to die suddenly is better, and for children to be established (married) before then. Important to be cared for by family
Sri Lanka					
Sri Lanka‐Patients					
(Perera et al. 2015) Ceylon Journal of Otolaryngology 2015	To identify aspects of a good death for patients	Ratnapura, Sri Lanka	Patients with life‐limiting illness (cancer and non‐cancer) attending ear, nose and throat, palliative care and oncology units (*n* = 42)	Qualitative Interviews	Preferred place of death was home 1. Good family relationships. 2. Good relationships with healthcare staff. 3. Being unaware of death. 4. Fighting the illness. 5. To die in a favorite place. 6. Faith. 7. Hope. 8. Dignity. 9. Freedom from pain and physical symptoms. 10. Not being a burden. 11. To have a sense of control. 12. Completion of life. 13. Appreciating others. 14. To not prolong life. 15. To contribute to others
Sri Lanka‐Healthcare Professionals					
(Chang et al. 2021) BMC Medical Ethics 2021	To investigate knowledge and attitudes about end of life and good death among doctors in Sri Lanka	Sri Lanka	Doctors (mostly junior) (*n* = 450)	Quantitative Nationwide survey	76% knew that a doctor should inform a patient of a terminal diagnosis, 66% favored doing so 74% felt knowing diagnosis would reduce a patient's uncertainty and anxiety, and 88.5% felt patients would adjust to the diagnosis. 85% worried about depressing the patient and 10.5% felt no benefit in telling patient. 11% felt family should be told the bad news. 51.5% of doctors felt that they would not place a young patient with metastatic cancer on a ventilator in order to prolong life by a few weeks Regarding a good death, 75% or more of doctors supported that patients should know when death is coming; have control, dignity and privacy; symptom control; choice regarding place and time of death and who is present at the time; access to information, spiritual and emotional support; access to hospice care in any location; have time to say goodbye; not prolong life unnecessarily and be able to issue advanced care directives which are respected. 58% felt it was important to have lived a wholesome and virtuous life, and only 32% felt that living a long life mattered (personal communication).
Bhutan					
Bhutan‐Healthcare Professionals					
(Laabar et al. 2022) PLOS Global Public Health 2022	To explore views of healthcare professionals in Bhutan regarding palliative care delivery	Tertiary, regional, district, community and traditional hospitals; primary health centers; Bhutan	Healthcare workers—Doctors, nurses, specialists, pharmacists, physiotherapists, health assistants, traditional medicine doctors (*n* = 63)	Qualitative Focus groups Interviews	Dying at home provides dignity. Patients should be aware of diagnosis and prognosis, and be supported on receiving this information. Involving *Drungtshos* would help with psychological and spiritual support, and can incorporate traditional medicine for these purposes.

In the quality appraisal, some studies were limited by failing to discuss the language used for data collection and analysis, and the translation processes used. Most of the qualitative studies did not discuss reflexivity, data saturation, or how the data presented in papers were selected. However, all studies were felt to be relevant to practice in terms of deepening understanding, identifying areas for further research, and building evidence for the generation of policies. Appraisal of quantitative studies revealed that most studies collected data directly from participants in a uniform manner. Purposive and convenience sampling methods were sometimes used instead of random selection methods, and questionnaires were often not provided. Given the lack of validated and well‐established tools for the region, many studies adapted existing questionnaires, with varying forms of validity and reliability‐checking. All qualitative and most quantitative studies were judged to be of moderate or high quality.

### 4.1 Synthesis

Through the narrative synthesis, four major themes emerged from the data. These were *Mutual care and connection support a continued sense of self*; *Freedom to choose—privilege or burden?*; *Opportunities in the last days*; and *After death matters*. These themes are discussed in turn.

For coherence and readability, the nationality of participants will be reported with the results of relevant studies cited, noting, however, that these are not necessarily broadly representative.

#### 4.1.1 Theme 1: Mutual Care and Connection Support a Continued Sense of Self

This theme illustrates how giving and receiving care supports patients to have an ongoing sense of personhood. For patients with advanced illness, contributing to the welfare of “those who matter” appeared to facilitate a sense of dignity and value. In parallel, receiving care from family, healthcare workers and through spiritual practices was key for this.

##### 4.1.1.1 Giving Helps Patients Maintain a Sense of Self

The importance of patients maintaining a strong sense of self through having purpose and relevance was evident among patients and healthcare workers and manifested in various forms (Chang et al. 2021; Coenen et al. 2007; Doorenbos et al. 2006; Perera et al. 2015; Pinto et al. 2019; Raju and Krishna Reddy 2018). These included not being a burden and ensuring family welfare by engaging in tasks of preparation and completion.

Across countries, patients highlighted the need to not burden family (Raju and Krishna Reddy 2018) and other patients, (Wajid et al. 2021) be it physically, financially, (Chacko et al. 2014; Perera et al. 2015), or through expressing emotional distress (Wajid et al. 2021). Being a burden was viewed as a “harm” to others (Wajid et al. 2021), and avoidance of being a burden was seen as a form of dignity (Earnest and Gnanadurai 2022; Zafar et al. 2016). Being physically and mentally able (Perera et al. 2015) and independent was valued (Wajid et al. 2021) among patients as well as community members (Joarder et al. 2014). Furthermore, being able to serve others resulted in patients achieving a sense of fulfillment (Wajid et al. 2021).

In the studies among Indian, Pakistani, and Bangladeshi patients, the perceived merits of preparation and completion before death were apparent (Chittem et al. 2022; Joarder et al. 2014; Nadeem et al. 2017; Raju and Krishna Reddy 2018; Simha et al. 2013). This involved having a will that outlined wishes for possessions, plans for their children's futures, and rituals following death (Nadeem et al. 2017) and practicing forgiveness (Simha et al. 2013). Great value was placed on being able to fulfill responsibilities toward family (Chittem et al. 2022; Simha et al. 2013), in the form of arranging financial security for spouses and children (Chittem et al. 2022; Nadeem et al. 2017; Raju and Krishna Reddy 2018), and knowing that children were well established, this is, married (Chittem et al. 2022; Joarder et al. 2014; Raju and Krishna Reddy 2018) having their own families and occupations (Chittem et al. 2022). For some Indian women with terminal illnesses, their responsibilities extended to having the status of a *sumangali* (being a married woman when they died), in line with expected cultural norms, and thus being able to accept death with more ease (Chittem et al. 2022). Of note, preparation and completion were not prioritized in a study among Sri Lankan patients, who ranked this 12 of 15 indicators (Perera et al. 2015) and another study noted nearly a fifth of Pakistani patients on dialysis felt it was unimportant or somewhat unimportant to prepare and plan in case of death (Saeed et al. 2020).

Studies in Indian, Pakistani, and Sri Lankan healthcare workers corroborated the importance of preparatory acts for patients, describing acts such as writing letters, leaving money and jewelry for loved ones (Pinto et al. 2019), saying goodbye, forgiving others, and resolving legal issues, property matters, and family concerns. Family concerns included children's marriages and financial security in the form of heirlooms (Chang et al. 2021; Nadeem et al. 2017; Pinto et al. 2019) It was felt that acceptance of death enabled patients to voice their wishes at the end of life and make necessary arrangements, offering a sense of control. If family were not able to provide support, one study reported that Indian healthcare workers would give time and effort to assist with weddings, reunions with family, deliver letters, and fulfill lifelong wishes (Pinto et al. 2019).

These findings illustrate the importance of maintaining a sense of self as death approaches, and the critical action of giving to promote this feeling. This was honored and supported by healthcare professionals.

##### 4.1.1.2 Receiving Care Through Connection Enhances a Sense of Self

In addition to giving, it was important for patients to receive care through human connection (with family and healthcare workers) and spiritual connection, thus further consolidating a sense of self.

###### 4.1.1.2.1 Receiving Care Through Human Connection

Fostering human connection was an important priority identified in several studies. While supporting others, patients concurrently receiving support from family and healthcare staff was a significant part of what mattered at the end of life for patients (Chacko et al. 2014; Zafar et al. 2016).

In terms of family support, Pakistani studies showed that, for patients with cancer, family was integral to the care process, through knowledge of the medical condition, treatments, and decision‐making (Saeed et al. 2020; Zafar et al. 2016). Patients wished for family to be aware of their diagnosis and treatments, and some preferred these conversations to be had with family exclusively (Zafar et al. 2016). In studies from India and Pakistan, family decision‐making was sought if a patient lost their capacity (Chacko et al. 2014; Saeed et al. 2020). Indian patients felt more comfortable discussing end‐of‐life preferences with their family rather than their healthcare team (Chacko et al. 2014). The involvement of family in care was supported by Indian nurses who felt it was necessary and a source of dignity for patients (Doorenbos et al. 2006). Interestingly however, a discrete choice experiment rated contact with family tenth out of 13 indicators of what “mattered to patients at the end of life,” as rated by bereaved proxy caregivers in India (Sepulveda et al. 2022).

In addition to medical information, patients tended to look to families for emotional and social support (Chacko et al. 2014; Perera et al. 2015; Saeed et al. 2020) including discussing their fears (Chacko et al. 2014) and anxieties (Raju and Krishna Reddy 2018), and they valued being cared for by family (Joarder et al. 2014). Caregivers in India described the importance of psychological strength and peace for patients (Namdul 2021; Ramalingam and Ganesan 2019; Sudhakar et al. 2021), attributed to an acceptance of and preparation for death (Sudhakar et al. 2021). Families engaged in spiritual practices, described below, as a means of strengthening psychological well‐being for patients.

In return, the education and support of family were also prioritized by patients and healthcare workers. (Coenen et al. 2007) This involved knowing that family was also prepared for impending death (Perera et al. 2015), educating family on the illness (Chacko et al. 2014), and knowing that time has been spent with family (Perera et al. 2015).

According to healthcare workers, patients across countries valued receiving psychological and emotional support (Saeed et al. 2020; Zafar et al. 2016) and kindness (Wajid et al. 2021). Indian healthcare workers also recognized patients' need for connection with family (including through home visits), partners (including through sexual intimacy) and healthcare workers (Pinto et al. 2019). Healthcare workers provided support by discussing anxieties (Chang et al. 2021; Doorenbos et al. 2006), being present (Coenen et al. 2007; Pinto et al. 2019), and providing physical touch, thereby maintaining faith and hope and reducing distress (Coenen et al. 2007).

###### 4.1.1.2.2 Supporting Spiritual Connection

Connection was also sought through spiritual means—particularly via rituals. These were practiced independently or with the support of others, and enabled the patient's spiritual self to be recognized.

Spiritual support for patients was emphasized in many studies (Chacko et al. 2014; Devakirubai and Gnanadurai 2013; Nadeem et al. 2017; Zafar et al. 2016) and was associated with peace and security (Simha et al. 2013). In India, to be able to counteract previous karma through *poojas*, to maintain faith by looking at photographs of God (Simha et al. 2013) and to pray and chant mantras (Raju and Krishna Reddy 2018) were important for patients. Sri Lankan patients valued feeling protected by good karma (Perera et al. 2015). Some Indian patients also reported a need to discuss spiritual needs with healthcare workers (Chacko et al. 2014; Devakirubai and Gnanadurai 2013). In contrast, in the discrete choice experiment by Sepulveda et al., spiritual needs were rated 11th of 13 (although still higher in Indian participants than in the UK (5.7% vs. 3.1%)) (Sepulveda et al. 2022).

In India, examples of important rituals conducted by family included oil bathing, feeding holy water or milk, or water mixed with mud. Family was thereby directly involved in creating a peaceful death (Ramalingam and Ganesan 2019). Family would perform the last rites as a duty (described further in *Opportunities in the last days*).

Across countries, healthcare workers too recognized the value of encouraging spiritual practices through various rituals as a means of emotional and psychological support (Chacko et al. 2014; Chang et al. 2021). In Bhutan, patients were allowed to light lamps and incense, and family and spiritual leaders would perform traditional rituals (Laabar et al. 2022). In India, in addition to facilitating rituals performed by others (Coenen et al. 2007; Doorenbos et al. 2006), nurses engaged in praying themselves (Coenen et al. 2007). Furthermore, traditional practices such as chanting *Bhajams* (prayers) and yoga meditation were performed (Doorenbos et al. 2006).

The relevance of spiritual leaders was also illustrated, including pastors in India (Doorenbos et al. 2006) and Buddhist Lamas and *Drungtshos* in Bhutan (Laabar et al. 2022). *Drungtshos* recognized their ability to contribute to psychological and emotional well‐being by incorporating aspects of traditional medicine such as acupuncture, yoga, massage, and meditation, as well as addressing spiritual aspects such as faith and karma and chanting mantras (Laabar et al. 2022).

These reciprocal acts of care between patients and families, supported by healthcare workers, appear to contribute significantly to conceptions of a good death in South Asia.

#### 4.1.2 Theme 2: Freedom to Choose—Privilege or Burden?

Opinions surrounding patients' choice to participate in care, and in what manner, varied. Although patient participation in care was generally valued, some preferred not to participate. Choice appeared as a privilege in circumstances where uncontrolled pain or financial distress precluded options. For some patients and healthcare workers, the challenges of choice were also observed regarding decisions that could artificially prolong life.

##### 4.1.2.1 Participation in Care Is a Choice

The evidence supports that patients and healthcare workers agree that patients should have the opportunity to participate in their care (Chacko et al. 2014; Chang et al. 2021; Chittem et al. 2022; Doorenbos et al. 2006; Perera et al. 2015; Raju and Krishna Reddy 2018; Saeed et al. 2020; Sepulveda et al. 2022; Zafar et al. 2016). However, in the face of poor prognosis, while some support patients being active participants in decision‐making by accessing knowledge and expressing preferences (Chang et al. 2021; Laabar et al. 2022; Pinto et al. 2019; Raju and Krishna Reddy 2018; Saeed et al. 2020; Sudhakar et al. 2021; Zafar et al. 2016) others support rescinding responsibility to family and healthcare providers. (Chacko et al. 2014; Chang et al. 2021; Perera et al. 2015; Saeed et al. 2020; Sudhakar et al. 2021; Zafar et al. 2016).

###### 4.1.2.1.1 Sharing Information and Involvement in Decision‐Making

Patients from several countries expressed a desire for access to information about their illness, treatment options (including withdrawal of care) and prognosis, along with discussions regarding quality of life (Chacko et al. 2014; Perera et al. 2015; Raju and Krishna Reddy 2018; Saeed et al. 2020; Sepulveda et al. 2022; Zafar et al. 2016).

The ability to express wishes and make decisions was important to patients. Sri Lankan patients expressed the significance of being listened to and having one's values respected (Perera et al. 2015). In Pakistan, this was reflected by patients highlighting the importance of advance care planning (Saeed et al. 2020). Indian patients described suffering with burdensome treatment, their wish to decline active treatment and hospital admissions, and instead choose where to spend their time (Chittem et al. 2022).

Healthcare workers echoed similar sentiments, with Indian nurses emphasizing the benefits of providing presence, showing active interest, and encouraging open discussions regarding death (Doorenbos et al. 2006). Sri Lankan doctors agreed that patients should know when and how death would occur (Chang et al. 2021), and nurses in India highlighted the need for clear communication to maintain dignity and reduce distress (Doorenbos et al. 2006) although discussing quality of life appeared less key for Indian healthcare workers in one study (Chacko et al. 2014).

###### 4.1.2.1.2 Discussing Diagnosis and Prognosis

Regarding attitudes to disclosure of diagnosis and prognosis, Indian and Pakistani patients acknowledged that knowing this information mattered (Raju and Krishna Reddy 2018; Saeed et al. 2020) and a healthcare worker should discuss this with them (Zafar et al. 2016) Some Indian family caregivers also recognized that knowledge of disease and prognosis was empowering and enabled preparation for patients, whereas being unaware led to uncertainty and distress (Sudhakar et al. 2021). Disclosure was also generally considered important by healthcare workers, with timely, accurate information felt to be of value, combined with support to absorb the information (Chang et al. 2021; Laabar et al. 2022; Pinto et al. 2019). In a survey of Sri Lankan doctors, most felt that a negative reaction would occur with breaking bad news, although this would be temporary and ultimately lead to reduced uncertainty for patients (Chang et al. 2021). These findings suggest that the need for information, including to be aware of diagnosis and prognosis, is respected among South Asian patients, relatives, and healthcare workers.

However, nearly a quarter of patients on dialysis studied in Pakistan by Saeed et al. did not place great emphasis on knowing their prognosis, with nearly a fifth feeling that detailed medical information was not of importance (Saeed et al. 2020). Another Pakistani study among cancer patients by Zafar et al. noted that although around three‐quarters of patients wanted updates about their progress with treatment, and at least 89% wanted a general understanding of the possible effectiveness of their treatments, only 60% wanted detailed explanations about chances of survival (Zafar et al. 2016). Being less likely to want a detailed prognosis was associated with patients being over 60 years old, female, or of lower socioeconomic status. Nearly a third of the study sample preferred healthcare workers to discuss such matters with their families instead. Moreover, nearly half the sample felt a healthcare worker was justified in withholding negative information from them if there were concerns of a negative impact on mental health, hope, or if their family requested it. Some Sri Lankan patients preferred to leave their physician to make decisions for them and tended to prioritize being unaware of the disease, fighting the illness, and living with hope and faith (Perera et al. 2015). Additionally, certain family members in India also felt that being unaware of the truth allowed patients to retain hope, knowing that the truth would be distressing (Sudhakar et al. 2021). Among Sri Lankan doctors, a minority felt disclosure of bad news was not beneficial to the patient and family should be told instead (Chang et al. 2021). An Indian study noted that clinicians tended to discuss prognosis with families preferentially, given concerns about negative psychological effects on patients (Chacko et al. 2014).

##### 4.1.2.2 The Opportunity to Choose Requires Freedom From Suffering

There was widespread agreement transnationally among patients and healthcare workers that pain and symptom control were critical for a good death (Chacko et al. 2014; Chang et al. 2021; Earnest and Gnanadurai 2022; Perera et al. 2015; Saeed et al. 2020; Sepulveda et al. 2022; Simha et al. 2013; Wajid et al. 2021; Zafar et al. 2016). Specific symptoms other than pain and methods of symptom management were generally not discussed. However, Indian nurses described a holistic approach to managing pain, dyspnea, and nausea, using pharmacological and non‐pharmacological measures including yoga meditation (Coenen et al. 2007; Doorenbos et al. 2006).

In the absence of symptom control, death was the alternative for some (Chittem et al. 2022; Simha et al. 2013). In the context of experiencing uncontrolled pain, some wished to be able to end suffering through suicide or euthanasia (Chittem et al. 2022). Notably, Ramalingam et al. reported how families in rural India believed certain traditional rituals to be necessary for a peaceful and dignified end, performed partly to “end the suffering.” Such rituals included feeding holy water, or water mixed with mud to the dying relative (Ramalingam and Ganesan 2019).

Similarly, suffering resulting from financial stress was of great significance for 91% of patients surveyed in Chacko et al.'s study in India (Chacko et al. 2014). This study reported on the debilitating impacts of financial suffering, such as suicidal ideation, refusal of therapies, fear of abandonment, and high demand for financial assistance (86% of patients). The same study showed that 30% of healthcare workers placed similar importance on patients' financial status.

The freedom to make decisions and participate in one's care appear limited in the face of extreme physical and/or financial suffering.

##### 4.1.2.3 The Challenges of Choice

Among Indian and Pakistani patients, surveys revealed mixed opinions concerning the artificial prolongation of life, including the use of mechanical ventilation, cardiopulmonary resuscitation, and artificial hydration and nutrition. (Chacko et al. 2014; Devakirubai and Gnanadurai 2013; Saeed et al. 2020; Zafar et al. 2016) Hindu patients emphasized integrating their spiritual beliefs regarding rebirth into decisions about the artificial prolongation of life (Chacko et al. 2014). Nearly half of patients on dialysis surveyed in Pakistan prioritized comfort over the quantity of life when required to choose between them (Saeed et al. 2020) whereas a third were unsure and a fifth preferred the prolongation of life. Of 42 Sri Lankan patients with life‐limiting illnesses who were interviewed, 29 regarded maximal life‐prolonging therapy as part of a good death (Perera et al. 2015). Nearly a third of patients attending a geriatric clinic in India wanted to receive all possible treatments while they were dying (Pany et al. 2018), and almost all patients with cancer in Pakistan wanted all life‐prolonging and palliative care concurrently until death (Zafar et al. 2016). To die in old age, naturally and without unnecessary suffering, was also valued by members of the public in Pakistan in an ethnographic study. (Ali 2020).

Most Sri Lankan doctors supported allowing a natural death without unnecessary prolongation, agreeing that to have lived a long life was not a prerequisite for a good death. Nevertheless, in a hypothetical case scenario, half agreed they would mechanically ventilate a young doctor with metastatic cancer to prolong life by a few weeks (Chang et al. 2021).

Despite the quality of life being important to patients (Sepulveda et al. 2022), choosing between quality and quantity of life appears difficult and perhaps an unreasonable expectation.

#### 4.1.3 Theme 3: Opportunities in the Last Days

A safe place of death was important. There was widespread agreement that death would ideally be at home (Chacko et al. 2014; Chittem et al. 2022; Devakirubai and Gnanadurai 2013; Pany et al. 2018; Perera et al. 2015; Raju and Krishna Reddy 2018; Saeed et al. 2020) and preferably in the presence of family (Chittem et al. 2022; Earnest and Gnanadurai 2022; Joarder et al. 2014; Pany et al. 2018; Perera et al. 2015; Pinto et al. 2019). However, home was not always the preferred place to die (Zafar et al. 2016). Religious views were noted to influence what circumstances mattered during the dying process, and performance of last rites was essential for families.

A location associated with peace, comfort, and security was valued by patients (Perera et al. 2015; Sepulveda et al. Wajid et al. 2021), which for some Indian patients was a hospice (Wajid et al. 2021). Dying where one belongs was also recognized as important by members of the public in Pakistan (Ali 2020) and healthcare workers in Bhutan felt this was important for dignity (Laabar et al. 2022). They also valued a peaceful environment. Indian nurses described singing songs to create a caring environment (Coenen et al. 2007). To have choice around where death occurred and who was present at the time was also felt to be key according to Sri Lankan doctors (Chang et al. 2021).

However, location and environment at the time of death were not important to everyone. Sepulveda et al. showed that place of death was ranked as a low priority by bereaved Indian caregivers, whereas receiving treatment in a clean safe space was ranked highly (Sepulveda et al. 2022). Likewise, Zafar et al. showed that only 42% of patients expressed a wish to die at home, knowing that some medical treatment may need to be foregone, with this number dropping to 19% in a survey 3 months later (Zafar et al. 2016). For Tibetan Buddhist caregivers in India, the state of mind at the time of death was of utmost importance. To be aware and focused was felt to be more impactful than external circumstances, including surroundings, in determining readiness to die (Namdul 2021). In Pakistan, patients and healthcare workers felt the timing of death mattered, but that time and place of death were predetermined by God (Nadeem et al. 2017).

Other factors important at the time of death included dying quickly and without awareness (Perera et al. 2015; Wajid et al. 2021), without pain (Namdul 2021; Perera et al. 2015; Wajid et al. 2021), in peace (Namdul 2021), and in old age (Pany et al. 2018). For community members in Bangladesh, how one died was a reflection of one's previous actions. Good deaths were considered given by God, whereas unnatural and violent deaths were due to bad karma (Joarder et al. 2014). Many Sri Lankan doctors also felt that to have lived a virtuous life was an aspect of a good death (Chang et al. 2021).

Families and healthcare workers considered the performance of last rites a critical part of a good death, a marker of respect and dignity. In Pakistan, the public considered last rites essential and involved ablution of the body, which was covered in a *Kafan* (white shroud), carried on a *charpoy* (woven bed), and included the funeral prayer *Namaz e Janaza* (Ali 2020). In Bhutan, relatives would read the Tibetan Book of the Dead to the patient (Namdul 2021). In India, last rites were performed by family using *tulsi* (basil), *Ganga Jal* (holy water), oil, *bindis*, *sindoor*, sandalwood, and reading the holy text, the *Bhagavad Gita*. The value placed on these rituals by healthcare workers is evident in them assuming the responsibility to perform these acts in the absence of family during the COVID‐19 pandemic. Crematorium staff in India described similar events during the COVID‐19 pandemic, where families would engage in paying large sums of money to them so that their deceased relatives would receive their last rites. Alternatively, another community member would perform the rituals, regardless of their caste or religion (Ghosh and Bk, 2024).

#### 4.1.4 Theme 4: After Death Matters

What happens after death appears to influence the actions of all key players in the lead‐up to death, shaped by the importance of leaving a legacy and religious beliefs.

Planning their legacy after death through a form of ongoing contribution was important to patients from several countries. This included being remembered for their service to others (Wajid et al. 2021) and by specifying their post‐death rituals (Nadeem et al. 2017). Legacy further extended to the bereaved family, with patients wanting to know that their families would do well following their death, highlighting that acts of completion and being in relationship with others would have a direct ongoing impact (Chittem et al. 2022; Perera et al. 2015; Raju and Krishna Reddy 2018; Simha et al. 2013). Healthcare workers also acknowledged this aspect in completion and preparation for death, giving patients the opportunity to leave a legacy behind after death. This could be in the form of leaving monetary gifts or letters, a will, or specifics of post‐death ceremonies (Nadeem et al. 2017; Pinto et al. 2019).

Beliefs regarding the afterlife impacted the actions of patients and families in India and Pakistan. Healthcare workers recognized that, for some patients in India, a belief that they would not see their families again after death led to the formation of suicide pacts with their spouses (Pinto et al. 2019). They also noted that the importance of performing last rites for families was embedded in a belief that, therefore, the soul would not return (Pinto et al. 2019). Indian families also felt that performing last rites enabled the person to go to Heaven, meet God, and their deceased ancestors (Ramalingam and Ganesan 2019).

The concept of rebirth also impacted people's actions and attitudes in Tibetan Buddhist communities in India. Caregivers felt that by being kind and reading Buddhist texts, they would accumulate good karma, which would influence their rebirth. This would enable a peaceful death and allow them to be prepared for the next life (Namdul 2021). Spiritual leaders in the Tibetan Buddhist tradition echoed these sentiments, speaking of dying with love and joy and viewing death as an opportunity to experience profound awareness and transcend the self. The state of mind at the time of death was influenced by past karma and would determine the next birth. These leaders expressed that contemplating death would strengthen the state of the mind at the time of death itself (Namdul 2021).

## 5 Discussion

### 5.1 Main Findings

This is the first review, to the best of our knowledge, that examines the concept of a good death within South Asia. Through collation and synthesis of the literature across different methodologies and nations, this review presents a coherent view of preferences and values around the end of life for South Asian people.

This synthesis illustrates the importance of relationships at the end of life in the South Asian context, and how this contributes to a sense of self. Contribution through acts of preparation and completion, including arranging post‐death matters, was critical for many patients. Patients sought and valued connection with their families and healthcare workers and through spiritual practices. Family was a central player in providing psychological support through spiritual acts and practical caregiving.

Our review highlights issues around choice. Choice was freeing for some and burdensome for others. In some instances, choice appeared limited due to more pressing issues such as poor pain control or financial difficulty. Some choices appeared difficult for all parties—such as balancing the need for comfort with a wish for prolongation of life.

To die feeling safe, without pain and in the presence of family was widely endorsed. Family again played a key role, performing last rites, thereby enabling a peaceful death and knowing that their loved ones would be protected after death. Throughout, what happens after death strongly influenced the thinking and actions of all those involved in the lead‐up to death and in the active dying process itself.

Generally, there was agreement between patients, families, and healthcare workers regarding many aspects of dying well, although potential sources of conflict centered around decisions regarding the patient's knowledge of their illness and treatment decisions regarding prolongation of life. It was also highlighted that healthcare workers may place less emphasis on discussing quality of life and finances than patients.

### 5.2 What This Study Adds

When Zaman et al. performed their “review of reviews” in 2021 on what it would take to die well, of the eight reviews concerning adults that clearly stated the countries from which the studies originated, only three studies were from South Asia (two from India, one from Bangladesh). Zaman et al. highlighted the concern that existing literature is skewed by the prevalence of research from high‐income settings with Western cultural norms. We conducted our review to bring attention to this gap and highlight emerging South Asian literature in this field.

In this current study, there is a predominance of studies from India, with limited evidence from the rest of South Asia, along with a previously noted absence of studies from certain countries in the region (Singh and Harding 2015). This finding is a call for research activity in these nations.

Despite the different cultural and religious settings across South Asia, our findings of the importance of symptom control, clear communication, cultural and spiritual rituals, management of distress, dying in a preferred place, receiving emotional support, and not being a burden for a good death largely mirrored those of Zaman et al. (Zaman et al. 2021).

However, there were differences found concerning autonomy around treatment decisions, not prolonging life unnecessarily, being aware of the great significance of the end‐of‐life process, and having the right to end one's own life. As acknowledged by Zaman et al. (2021) and as reflected in our findings, autonomy is multifaceted, dying in a preferred place is not always possible, decisions regarding comfort versus prolongation of life are challenging, and having awareness of dying is not preferred by everyone.

Other studies corroborate some of these differences. An integrative review of a good death in Western societies discusses the importance of “controlling dying,” particularly by dying at home (Cottrell and Duggleby 2016). The authors also highlighted the necessity for patients to accept death to allow a good death to occur. In our study, dying at home was not always the preferred option, with some patients finding security in a hospice or hospital setting. Furthermore, in our findings, knowledge of impending death was not always considered necessary by patients, caregivers, or healthcare workers. Finally, in stark contrast to our findings, Cottrell and Duggleby (2016) placed little emphasis on the role of family or religion for a good death, yet did note a cohort of rural Canadians that greatly valued connection with family and community (Wilson et al. 2009). It may be that the dominant narrative of a good death, including in Western societies, represents only certain voices. Meanwhile, there may be subsections of such populations that hold values similar to those we have illustrated in our review. The dominance of a particular narrative in palliative care is supported by a Danish ethnographic study, which illustrates the differences in needs and care between two patients of differing socioeconomic status (Aamann and Dybbroe 2024). Globally, and within specific populations, it is possible that more prominent voices shape what a good death should be.

Here, we discuss two aspects of the end‐of‐life pertinent to South Asia, namely participation in decision‐making and the role of religion and spirituality.

In our review, patient views on participating in care and making decisions ranged from significant involvement to no involvement at all, with some preferring family to take over this responsibility. We observe that there are degrees of detail in what is disclosed to patients in terms of diagnosis, prognosis, and treatments. There are also levels of comfort that patients have with the amount of detail about various topics, for example, most patients in the study by Zafar et al. wanted general ideas about treatment success, but fewer were comfortable with detailed statistics about chances of survival (Zafar et al. 2016). Our results support other data from South Asia, where among patients with advanced cancer in India, Sri Lanka, and Bangladesh, awareness of cancer stage and prognosis is very low (Ozdemir et al. 2022, 2022; Satija et al. 2022) and most patients play no role in decision‐making, although for the majority, and especially Sri Lankan patients, this appears to be concordant with their preferences (Semra Ozdemir et al. 2021).

Given the layers of nuance surrounding information‐giving to patients, we suggest that tailoring information needs to a patient in the context of their overall life goals, and with the assistance of their social network, can be viewed as an effort to uphold that person's belief of how their life should be lived. This resonates with Stonington's ethnographic work on what is considered a good death in Northern Thailand, where he illustrates the layers and states of “knowing” that a patient can choose to have regarding their illness (Stonington 2020). The different forms of participation in care deserve further recognition, particularly when designing processes around breaking bad news and advanced care planning in South Asia, so that these preferences are central rather than viewed as obstacles.

The impact of poverty on palliative care in South Asia is evident in its effect on decision‐making (Rajagopal and Venkateswaran 2004). In South Asia, 10.9% of people live on less than $2.15 a day, and 50% of urban populations live in informal settlements (World Health Organisation 2024). Inequity in the provision of palliative care in low‐ and middle‐income countries is well documented, resulting in preventable SHS (Knaul et al. 2018). Of the total number of people who die annually with SHS in low‐ and middle‐income countries, 63% of such deaths with SHS are avoidable (Knaul et al. 2018).

We raise two particular consequences of poverty on decision‐making. First, a lack of options poses a threat to decision‐making. Even in high‐income countries, quality of care available and access to care is seen to affect decision‐making (Higginson et al. 2017). In our review, Sepulveda et al. showed that “place of death” was not ranked highly as an indicator for a good death in India, possibly because it was not perceived as a choice (Sepulveda et al. 2022). Similarly, Zafar et al. showed that most Pakistani cancer patients did not opt to die at home, and over 3 months, over half of patients who had originally wished to die at home had changed their minds, possibly due to a lack of community palliative care services or free home care (Zafar et al. 2016).

We also found that uncontrolled pain and severe financial distress can lead to patients seeing death as the only option remaining. This raises the uncomfortable but necessary question of whether the current approach to end of life in South Asia is adequate, practical, and effective. This also causes us to question whether a good death is a concept that can be considered when severe pain and financial instability erode the will to live. The freedom to make decisions and participate in one's care appears limited in the face of extreme suffering. Current evidence suggests a discrepancy between patients and healthcare workers in recognizing these issues. There is a clear need to find cost‐effective solutions to the lack of access to palliative care, particularly symptom relief. The Lancet Commission on Palliative Care and Pain Relief has proposed an “Essential Package,” detailing medicines, equipment, and human resources needed, as a feasible, minimal cost option in the delivery of palliative care in resource‐poor settings (Knaul et al. 2018). Its implementation could be assessed as a step toward addressing this problem. However, the Commission notes that access to morphine and systems to provide formal social supports should be simultaneously addressed to ensure adequate palliative care delivery. These issues remain works in progress in many settings. Adaptation of models such as the Kerala “Neighborhood Network” model, which utilizes existing community networks to enable palliative care delivery, would also need to be considered and adapted for other South Asian settings (Kumar 2007).

Second, the impact of poverty on decision‐making raises difficult ethical questions. Perhaps due to limitations of choice, two surveys revealed that some patients wished to receive all possible treatments until death (Pany et al. 2018; Zafar et al. 2016). The treatment burden and financial status of these patients are unknown. This scenario forces uncomfortable ethical debate. What would be considered standard care (adequate screening, early detection and delivery of curative or life‐prolonging treatments) in high‐income countries may not be available or accessible in low‐income settings, resulting in certain potentially curable diseases thus being deemed fatal (Hadler and Rosa 2018; Rajagopal and Venkateswaran 2004). How does this lack of appropriate and timely care influence the practices of healthcare professionals, some of whom provide potentially inappropriate or ineffective treatments at the end of life? Would not available resources be better spent on curative/disease‐modifying treatments for those with early‐stage disease? (Hadler and Rosa 2018). Is it just to continue ineffective treatments at the end of life for conditions that may not have been managed optimally in the early stages, resulting in catastrophic health expenditure for families? (World Health Organisation 2023). Providing ineffective treatments for late‐stage disease is not congruent with holistic, person‐centered care. Yet, how do doctors interpret and practice beneficence and non‐maleficence in these challenging contexts? Research regarding the practical ethics of palliative care in low‐ and middle‐income settings is lacking and deserves further exploration (Schofield et al. 2021).

Our findings suggest that death retains a spiritual dimension, deeply embedded in networks of relationships, in South Asia. The consideration of the afterlife remains a key aspect of death in many religions (Sallnow et al. 2022). In our study, the impact of religion on various facets of a good death was prominent—particularly the influences of Islamic and Buddhist beliefs on the dying process, post‐death rituals, and the afterlife. The importance of such religious beliefs was evident through the emphasis on spiritual practices, performance of last rites, and—for Buddhists—the state of mind at the time of death.

As seen in our study, belief in karma and rebirth caused some Hindus to decline artificial prolongation of life, as lengthening or shortening life artificially can interfere with the karmic process and result in accumulating negative karma, which can affect subsequent rebirths (Thrane 2010). Although not apparent from the data in our review, there may be further impacts from these spiritual frameworks that would be beneficial to explore. These include guilt and fatalism from believing that one's suffering is a result of previous negative actions; taking personal responsibility to collect merit for the next life (Keown and Keown 2013); believing suffering is needed to pay off karmic debts, thereby declining analgesia; having a clear mind filled with good thoughts at the time of death, again leading to declining analgesia and sedation (Thrane 2010); and the impact of Islamic beliefs on patient involvement in care. As necessary as it is to understand these beliefs, they are only some of the many factors that interplay at the end of life—which may explain the need for some patients to want all possible treatments until death or to request assisted dying in the face of severe pain.

Our study also highlights the value of incorporating spiritual and traditional practices and spiritual leaders, when addressing pain and psychological and emotional distress. Certain spiritual practices have been identified as useful for people of any religious belief to adopt, such as living in the present moment (Masel et al. 2012). Future research can be directed at exploring how existing religious beliefs and spiritual leaders may be integrated into palliative care systems in South Asia, as a means of seamlessly providing psychological, emotional, and spiritual support.

### 5.3 Limitations

Aspects of the review process such as screening articles, extracting data, and initial coding were carried out by one reviewer with associated potential for bias. However, in the final round of selection for studies, the review was conducted by two authors with a third to adjudicate any disagreements. In addition, the quality appraisal process was also performed by two authors and used tools specific to the study type, adding to the strength of quality appraisal in this study. In addition, the analysis process was conducted in conjunction with all authors, further increasing the rigor of this review.

While we sought studies from all South Asian countries, there was a lack of studies on this subject from Nepal, Afghanistan, and the Maldives meaning that our overall understanding of a good death in South Asia is incomplete.

This review has limited itself to synthesizing empirical studies. We acknowledge there are many other sources of knowledge regarding this topic, from philosophy, religion, ethics, and sociology. Importantly, we have included perspectives of members of the public, spiritual leaders, and crematorium staff, which have been noted to be lacking in other reviews (Zaman et al. 2021).

## 6 Conclusions

This work is an effort to lessen the gap in palliative care research between high‐income and low‐income settings, and provides an initial overview of important aspects of end‐of‐life care in South Asia from the perspective of patients, families, healthcare workers, and members of the public. Family involvement, spiritual rituals, reciprocity of care, difficulties with choice, performance of last rites, and after‐death matters were prominent at the end of life. For a more complete picture, further data are required from most South Asian countries.

Overall, our findings suggest that the South Asian perspective of a good death shares many similarities with the Western perspective, with some important differences concerning decision‐making, prolongation of life, prognostic awareness, and wanting to end one's life, moderated by culture, poverty, and religion.

We support policies regarding palliative care in South Asia to account for the degrees of participation in decision‐making practiced by patients. Further efforts are required to provide good symptom management, thereby expanding opportunities for patient participation in care. Future research should investigate the interplay between poverty, decision‐making, and ethical implications for end of life in South Asia, and how spiritual beliefs and practices can be incorporated into palliative care. These findings are intended to generate discussion and ideas for developing palliative care in a manner compatible with South Asian needs and culture.

## Disclosure

Clinical resources: Diversity in Dying: Death across Cultures. Diversity in Dying: Death across Cultures—Nursing Care at the End of Life (geneseo.edu).

## Conflicts of Interest

The authors declare no conflicts of interest.

## Supporting information


Data S1.

## Data Availability

The data that support the findings of this study are available from the corresponding author upon reasonable request.
