# Supplementary material for: What Is a Good Death in South Asia? A Systematic Review and Narrative Synthesis
Source: J Nurs Scholarsh. 2025 Mar 18;57(4):653–77. doi: 10.1111/jnu.70002 (PMC12241761; doi:10.1111/jnu.70002)
Supplement: Supplementary file 1 — Data S1. [file JNU-57-653-s001.docx]

**Ovid MEDLINE(R) and Epub Ahead of Print, In-Process, In-Data-Review & Other Non-Indexed Citations and Daily**

| 1. | (good adj2 (death or deaths or dying or end-of-life)).mp. |
| --- | --- |
| 2. | (quality adj2 (death or deaths or dying or end-of-life)).mp. |
| 3. | (digni* adj2 (death or deaths or dying or die or end-of-life)).mp. |
| 4. | (undignified adj1 (death or deaths or dying or end-of-life)).mp. |
| 5. | ((die or dying) adj1 well).mp. |
| 6. | ((peace* or successful*) adj1 (death or deaths or dying or die or end-of-life)).mp. |
| 7. | (desire* adj3 (death or deaths or dying or die or end-of-life)).mp. |
| 8. | (bad adj1 (death or deaths or end-of-life)).mp. |
| 9. | ((prefer* or need* or perspective*) adj2 (die or death or dying or deaths or end-of-life)).mp. |
| 10. | 1 or 2 or 3 or 4 or 5 or 6 or 7 or 8 or 9 |
| 11. | exp Asia, Southern/ |
| 12. | (south asia* or southern asia* or southeastern asia* or south eastern asia* or southeast asia* or south east asia* or bangladesh* or bengal* or bangal* or sri lanka* or ceylon* or india* or bhutan* or maldives* or maldivian* or nepal* or pakistan* or afghan* or indian ocean or indian subcontinent).ti,ab,hw,kf,jw. |
| 13. | 11 or 12 |
| 14. | 10 and 13 |

**APA PsychInfo (Ovid)**

| 1. | (good adj2 (death or deaths or dying or end-of-life)).mp. |
| --- | --- |
| 2. | (quality adj2 (death or deaths or dying or end-of-life)).mp. |
| 3. | (digni* adj2 (death or deaths or dying or die or end-of-life)).mp. |
| 4. | (undignified adj1 (death or deaths or dying or end-of-life)).mp. |
| 5. | ((die or dying) adj1 well).mp. |
| 6. | ((peace* or successful*) adj1 (death or deaths or dying or die or end-of-life)).mp. |
| 7. | (desire* adj3 (death or deaths or dying or die or end-of-life)).mp. |
| 8. | (bad adj1 (death or deaths or end-of-life)).mp. |
| 9. | ((prefer* or need* or perspective*) adj2 (die or death or dying or deaths or end-of-life)).mp. |
| 10. | 1 or 2 or 3 or 4 or 5 or 6 or 7 or 8 or 9 |
| 11. | (south asia* or southern asia* or southeastern asia* or south eastern asia* or southeast asia* or south east asia* or bangladesh* or bengal* or bangal* or sri lanka* or ceylon* or india* or bhutan* or maldives* or maldivian* or nepal* or pakistan* or afghan* or indian ocean or indian subcontinent).mp. |
| 12. | exp south asian cultural groups/ |
| 13. | 11 or 12 |
| 14. | 10 and 13 |

**Embase Classic+Embase (Ovid)**

| 1. | (good adj2 (death or deaths or dying or end-of-life)).mp. |
| --- | --- |
| 2. | (quality adj2 (death or deaths or dying or end-of-life)).mp. |
| 3. | (digni* adj2 (death or deaths or dying or die or end-of-life)).mp. |
| 4. | (undignified adj1 (death or deaths or dying or end-of-life)).mp. |
| 5. | ((die or dying) adj1 well).mp. |
| 6. | ((peace* or successful*) adj1 (death or deaths or dying or die or end-of-life)).mp. |
| 7. | (desire* adj3 (death or deaths or dying or die or end-of-life)).mp. |
| 8. | (bad adj1 (death or deaths or end-of-life)).mp. |
| 9. | ((prefer* or need* or perspective*) adj2 (die or death or dying or deaths or end-of-life)).mp. |
| 10. | 1 or 2 or 3 or 4 or 5 or 6 or 7 or 8 or 9 |
| 11. | (south asia* or southern asia* or southeastern asia* or south eastern asia* or southeast asia* or south east asia* or bangladesh* or bengal* or bangal* or sri lanka* or ceylon* or india* or bhutan* or maldives* or maldivian* or nepal* or pakistan* or afghan* or indian ocean or indian subcontinent).ti,ab,hw,kf,jx. |
| 12. | exp maldives/ |
| 13. | exp South Asia/ |
| 14. | 11 or 12 or 13 |
| 15. | 10 and 14 |

**Global Health (Ovid)**

| 1. | (good adj2 (death or deaths or dying or end-of-life)).mp. |
| --- | --- |
| 2. | (quality adj2 (death or deaths or dying or end-of-life)).mp. |
| 3. | (digni* adj2 (death or deaths or dying or die or end-of-life)).mp. |
| 4. | (undignified adj1 (death or deaths or dying or end-of-life)).mp. |
| 5. | ((die or dying) adj1 well).mp. |
| 6. | ((peace* or successful*) adj1 (death or deaths or dying or die or end-of-life)).mp. |
| 7. | (desire* adj3 (death or deaths or dying or die or end-of-life)).mp. |
| 8. | (bad adj1 (death or deaths or end-of-life)).mp. |
| 9. | ((prefer* or need* or perspective*) adj2 (die or death or dying or deaths or end-of-life)).mp. |
| 10. | 1 or 2 or 3 or 4 or 5 or 6 or 7 or 8 or 9 |
| 11. | (south asia* or southern asia* or southeastern asia* or south eastern asia* or southeast asia* or south east asia* or bangladesh* or bengal* or bangal* or sri lanka* or ceylon* or india* or bhutan* or maldives* or maldivian* or nepal* or pakistan* or afghan* or indian ocean or indian subcontinent).mp. |
| 12. | exp south asia/ |
| 13. | exp maldives/ |
| 14. | exp Afghanistan/ |
| 15. | 11 or 12 or 13 or 14 |
| 16. | 10 and 15 |

**CINAHL Complete (EBSCOhost):**

# Query Limiters/Expanders

S5 S1 AND S4 Search modes - Boolean/Phrase Interface - EBSCOhost Research Databases

S4 S2 OR S3 Search modes - Boolean/Phrase Interface - EBSCOhost Research Databases

S3 (MH "Asia, Western+") Search modes - Boolean/Phrase Interface - EBSCOhost Research Databases

S2 ("south asia*" or "southern asia*" or "southeastern asia*" or "south eastern asia*" or "southeast asia*" or "south east asia*" or bangladesh* or bengal* or bangal* or "sri lanka*" or ceylon* or india* or bhutan* or maldives* or maldivian* or nepal* or pakistan* or afghan* or "indian ocean" or "indian subcontinent") Search modes - Boolean/Phrase Interface - EBSCOhost Research Databases

S1 ( good N2 (death or deaths or dying or "end-of-life") ) OR ( quality N2 (death or deaths or dying or "end-of-life") ) OR ( digni* N2 (death or deaths or dying or die or "end-of-life") ) OR ( undignified N1 (death or deaths or dying or "end-of-life") ) OR ( (die or dying) N1 well ) OR ( (peace* or successful*) N1 (death or deaths or dying or die or "end-of-life") ) OR ( desire* N3 (death or deaths or dying or die or "end-of-life") ) OR ( bad N1 (death or deaths or "end-of-life") ) OR ( (prefer* or need* or perspective*) N2 (die or death or dying or deaths or "end-of-life") ) Search modes - Boolean/Phrase Interface - EBSCOhost Research Databases

**Web of Science (Clarivate)**

#1

(((((((((TS=(good NEAR/2 (death or deaths or dying or end-of-life))) OR TS=(quality NEAR/2 (death or deaths or dying or end-of-life)))) OR TS=(digni* NEAR/2 (death or deaths or dying or die or end-of-life))) OR TS=(undignified NEAR/1 (death or deaths or dying or end-of-life))) OR TS=((die or dying) NEAR/1 well)) OR TS=((peace* or successful*) NEAR/1 (death or deaths or dying or die or end-of-life))) OR TS=(desire* NEAR/3 (death or deaths or dying or die or end-of-life))) OR TS=(bad NEAR/1 (death or deaths or end-of-life))) OR TS=((prefer* or need* or perspective*) NEAR/2 (die or death or dying or deaths or end-of-life))

#2

TS=(("south asia*" or "southern asia*" or "southeastern asia*" or "south eastern asia*" or "southeast asia*" or "south east asia*" or bangladesh* or bengal* or bangal* or "sri lanka*" or ceylon* or india* or bhutan* or maldives* or maldivian* or nepal* or pakistan* or afghan* or "indian ocean" or "indian subcontinent"))

#3

#1 AND #2

**Scopus (Elsevier)**

( TITLE-ABS-KEY ( ( good W/2 ( death OR deaths OR dying OR "end-of-life" ) ) OR ( quality W/2 ( death OR deaths OR dying OR "end-of-life" ) ) OR ( digni* W/2 ( death OR deaths OR dying OR die OR "end-of-life" ) ) OR ( undignified W/1 ( death OR deaths OR dying OR "end-of-life" ) ) OR ( ( die OR dying ) W/1 well ) OR ( ( peace* OR successful* ) W/1 ( death OR deaths OR dying OR die OR "end-of-life" ) ) OR ( desire* W/3 ( death OR deaths OR dying OR die OR "end-of-life" ) ) OR ( bad W/1 ( death OR deaths OR "end-of-life" ) ) OR ( ( prefer* OR need* OR perspective* ) W/2 ( die OR death OR dying OR deaths OR "end-of-life" ) ) ) ) AND ( TITLE-ABS-KEY ( ( "south asia*" OR "southern asia*" OR "southeastern asia*" OR "south eastern asia*" OR "southeast asia*" OR "south east asia*" OR bangladesh* OR bengal* OR bangal* OR "sri lanka*" OR ceylon* OR india* OR bhutan* OR maldives* OR maldivian* OR nepal* OR pakistan* OR afghan* OR "indian ocean" OR "indian subcontinent" ) ) )

**Google Scholar:**

((good AROUND(2) (death or deaths or dying or end-of-life)) OR (quality AROUND(2) (death or deaths or dying or end-of-life)) OR (digni* AROUND(2) (death or deaths or dying or die or end-of-life)) OR (undignified AROUND(1) (death or deaths or dying or end-of-life)) OR ((die or dying) AROUND(1) well) OR ((peace* or successful*) AROUND(1) (death or deaths or dying or die or end-of-life)) OR (desire* AROUND(3) (death or deaths or dying or die or end-of-life)) OR (bad AROUND(1) (death or deaths or end-of-life)) OR ((prefer* or need* or perspective*) AROUND(2) (die or death or dying or deaths or end-of-life))) AND ("south asia*" or "southern asia*" or "southeastern asia*" or "south eastern asia*" or "southeast asia*" or "south east asia*" or bangladesh* or bengal* or bangal* or "sri lanka*" or ceylon* or india* or bhutan* or maldives* or maldivian* or nepal* or pakistan* or afghan* or "indian ocean" or "indian subcontinent")
